# Oxidative Stress, Molecular Inflammation and Sarcopenia

**DOI:** 10.3390/ijms11041509

**Published:** 2010-04-12

**Authors:** Si-Jin Meng, Long-Jiang Yu

**Affiliations:** 1 Institution of Life Science and Technology, Huazhong University of Science and Technology, Wuhan 430074, China; E-Mail: msijin@wipe.edu.cn; 2 College of Health Science, Wuhan Institute of Physical Education, Wuhan 430079, China

**Keywords:** oxidative stress, chronic inflammation, signaling, sarcopenia, interventions

## Abstract

Sarcopenia is the decline of muscle mass and strength with age. Evidence suggests that oxidative stress and molecular inflammation play important roles in age-related muscle atrophy. The two factors may interfere with the balance between protein synthesis and breakdown, cause mitochondrial dysfunction, and induce apoptosis. The purpose of this review is to discuss some of the major signaling pathways that are activated or inactivated during the oxidative stress and molecular inflammation seen in aged skeletal muscle. Combined interventions that may be required to reverse sarcopenia, such as exercise, caloric restriction, and nutrition, will also be discussed.

## 1. Introduction

Sarcopenia refers to the decline in muscle mass and strength with age [1]. It leads to significant impairment in the ability to carry out normal daily functions and an increased risk of falls and fractures, and eventually leads to loss of independence [2]. It is estimated that approximately one-quarter to one-half of the population aged 65 and older is sarcopenic [3]. Given our rapidly aging population, research designed to better understand the development, progression, prevention, and treatment of sarcopenia is of substantial importance.

Age-related loss of muscle mass in rodents [4] and humans [5] occurs via the loss of muscle fibers and a decrease in the cross-sectional area of the remaining fibers. Daw *et al*. found that the muscles in 27-month-old Fisher 344 rats, had a 4%–5.6% loss of fibers when compared to those muscles in 12-month-old adult rats [4]. Additional oberservation was the atrophy of existing fibers [4]. Lexell *et al*., using whole muscle cross-sections from the vastus lateralis muscle obtained postmortem, reported that, by the ninth decade, ∼50% fewer type I and type II fibers were present compared with muscles from those 20 years old [5]. However, the cellullar and molecular mechanisms responsible for the fiber loss and atrophy remain elusive.

It has recently been suggested that oxidative stress, chronic inflammation, and mitochondrial dysfunction play important roles in age-related muscle atrophy [6]. The interaction of these factors may converge on several intracellular signaling pathways, affecting the balance between protein synthesis and breakdown, inducing apoptosis, which cause the primary pathology of significant loss of muscle mass.

**Figure 1.**
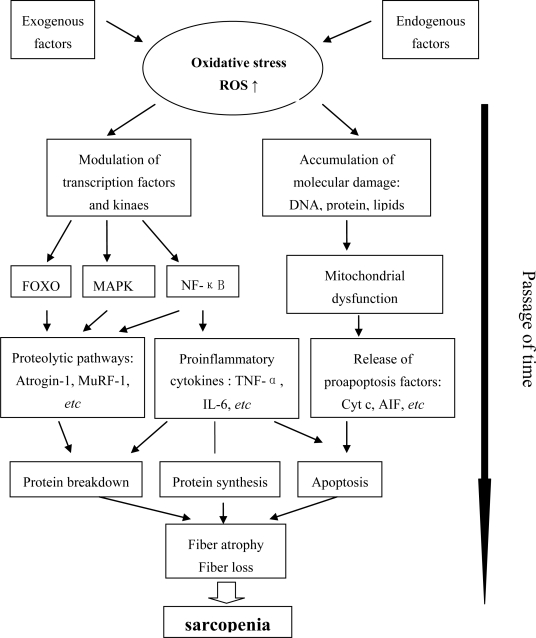
A schematic summary of proposed mechanisms by which oxidative stress and chronic inflammation could contribute to sarcopenia. Some major signaling pathways are activated or inactivated during the oxidative stress and chronic inflammation seen in aged skeletal muscle. The pathways are related to an imbalance of protein synthesis and breakdown, mitochondrial dysfunction, and apoptosis, leading to fiber atrophy and fiber loss, eventually to sarcopenia.

The purpose of this review is to discuss some of the major signaling pathways that are activated or inactivated during the state of oxidative stress and chronic inflammation seen in aged skeletal muscle. These pathways are related to mitochondrial dysfunction, an imbalance of protein synthesis and breakdown, and apoptosis, leading to fiber atrophy and fiber loss. A schematic summary of proposed mechanisms by which oxidative stress and chronic inflammation could contribute to sarcopenia, as seen in Figure 1. Combined interventions that may be required in order to attenuate and reverse sarcopenia, including exercise, caloric restriction, nutrition, and so on, will then be discussed.

## 2. The State of Oxidative Stress and Molecular Inflammation in Aging Muscle

### 2.1. Oxidative Stress

Oxidative stress is an imbalance in oxidant and antioxidant levels [7]. Aging has been shown to predispose skeletal muscle to increased levels of oxidative stress both at rest and during disuse atrophy [8], suggesting that oxidative stress has a role in mediating disuse-induced and sarcopenia-associated muscle loss. The state of oxidative stress seems to underlie the pathogenesis of chronic diseases that are accompanied by muscle wasting [9]. Low levels of serum/plasma antioxidant carotenoids are independently associated with low skeletal muscle strength and the development of walking disability [10]. Increased levels of chronic low-grade inflammation induced by oxidative stress have been shown to be detrimental to skeletal muscle in humans [11], as well as in animal models [8]. Under normal conditions there is a balanced and continuous degradation and resynthesis of skeletal muscle proteins. However, during the aging process and the resulting increased oxidative stress, this balance is disrupted [12]. This imbalance is perhaps because of blunted anabolic signaling and increased catabolic signaling, as discussed in detail below. The pathogenesis of sarcopenia is multifactorial and is attributed to oxidative stress, inflammation, endocrine changes, inactivity, and undernutrition. Many of the factors that have been implicated in sarcopenia do not act in isolation, and many of their causal pathways intersect or overlap in relation to oxidative stress.

### 2.2. Chronic Molecular Inflammation

An age-related disruption in the intracellular redox balance appears to be a primary causal factor in producing a chronic state of low-grade inflammation. Chronic molecular inflammation is considered as an underlying mechanism of aging and age-related diseases, and it may serve as a bridge between normal aging and age-related pathological processes [13]. The aging-related redox-sensitive transcription factor NF-*κ*B has been shown to induce inflammation. Age-related upregulation of the key players such as IL-6, TNF-α are mediated by NF-*κ*B [13]. Moreover, ROS (reactive oxygen species) also appear to function as second messengers for TNF-α in skeletal muscle, activating NF-κB either directly or indirectly [14]. In fact, increased oxidative stress and inflammation are well known to go hand in hand in many skeletal muscle–associated diseases. Chronic subclinical inflammation may be a marker of functional limitations in older persons across several diseases/health conditions [15]. There is one research showing a significant negative relationship between mixed-muscle and MHC (myosin heavy chain) protein synthesis rates and circulating concentrations of several markers of immune activation, including IL-6 and TNF-α [16], despite that how pro-inflammatory cytokines affect protein synthesis remains to be determined.

It has been shown that TNF-α is one of the primary signals that induce cellular apoptosis in muscle. Apoptosis and inflammation closely interact with oxidative damage and are all involved in age-related reduction in muscle mass and strength [6]. It has been suggested that inflammation may negatively influence skeletal muscle through direct catabolic effects or through indirect mechanisms (*i.e.*, decreases in GH and IGF-1 concentrations, induction of anorexia) [17]. The reduction of IGF-1 levels is associated with sarcopenia, frailty, and mortality. The anorectic effects of pro-inflammatory cytokines such as TNF-α are particularly interesting because nutrition is a crucial factor in the prevention of sarcopenia.

### 2.3. Mitochondrial Dysfunction

It has long been recognized that high levels of ROS can inflict direct damage on macromolecules, such as lipids, nucleic acids, and proteins. Mitochondria are a major source of ROS in skeletal muscle, and mitochondrial DNA may be especially susceptible to oxidative DNA damage [18]. Inflammation also impairs mitochondrial function in cardiac myocytes [19]. The accumulation of mitochondrial and nuclear DNA damage is thought to eventually compromise function, leading to the loss of myocytes [20]. Thus, the importance of mitochondrial dysfunction with age is related not only to the loss of the capacity to generate ATP but also to the activation of pathways that lead to the irreversible cell loss that is characteristic of neurodegeneration and sarcopenia [21].

Taken together, the effects of oxidative stress and molecular inflammation in skeletal muscle may lead to mitochondrial dysfunction, decreased protein synthesis, increased protein degradation, and apoptosis by activating or inactivating some major signaling pathways. These changes eventually lead to reduced muscle mass, as discussed below.

## 3. Signaling Pathways and Kinases Involved in Age-Related Muscle Atrophy

### 3.1. IGF-1/Akt/mTOR

Over the past few years, the IGF-1/PI3K/Akt (insulin-like growth factor 1/phosphatidylinositol 3-kinase/Protein kinase B) signaling pathways, which are responsible for regulating protein synthesis pathways, have been defined [22]. More recently, it has been shown that IGF-1 can also block the transcriptional upregulation of the key mediators of skeletal muscle atrophy, the ubiquitin-ligases MuRF1 (muscle RING finger-1 protein) and MAFbx (muscle atrophy F-box protein, also called Atrogin-1) [23]. Moreover, it is clear that over-expression of IGF-1 in muscle can protect against age-related sarcopenia [24].

Protein kinase B (PKB) /Akt is a serine/threonine kinase that signals via a wortmannin-sensitive pathway downstream of growth factor receptors by activating PI3K [25], the activity of which can be increased by IGF-1 receptor signaling, nutrients, and even muscle contraction. Akt plays a number of roles that may be important in sarcopenia. These roles include the suppression of apoptosis and protein degradation in skeletal muscle by promoting phosphorylation and inactivation of the pro-apoptotic protein Bad and FOXO (Forkhead box) transcription factors, respectively [26]. Latres *et al*. also showed that Akt phosphorylates FOXO transcription factors, thus inhibiting the expression of atrophy-related genes such as atrogin-1 and MuRF-1[27]. On the other hand, Akt activity also promotes protein translation via the inhibition of glycogen synthase kinase-3 and the activation of the mammalian target of rapamycin (mTOR) [28]. Therefore, Akt exerts its influence on both sides of the muscle protein balance equation.

The importance of intact mTOR signaling and of its downstream targets in linking nutritional and hormonal cascades to the regulation of cell size is well established [29]. Additionally, the downstream mTOR target, p70S6K, is strongly linked to muscle protein synthesis [30]. Thus, the Akt/mTOR/p70S6K signaling pathway is thought to play a role in the regulation of protein synthesis and skeletal muscle mass.

Considerable evidence also implicates age-related declines in muscle insulin-like growth factor activity in sarcopenia. Although there is evidence that aging muscle retains the ability to synthesize IGF-1, aging may be also associated with attenuation of the ability of exercise to induce an isoform of IGF-1 that promotes satellite cell proliferation [31]. Recent studies indicate an age-related decrease in both systemic and locally derived IGF-1, which may be responsible, at least in part, for the age-related decline in skeletal muscle structure and function, due to reduced activity of the Akt signaling pathway [32]. Moreover, aging muscle may be resistant to IGF-1, an effect that is reversed by exercise [33]. A large number of studies have suggested the implications of cross-talk between ROS [34], the proinflammatory cytokine TNF-α [35], and IGF-1 signaling in skeletal muscle, which is likely the underlying mechanism of resistance to IGF-1. Of course, further investigations need to be done to determine how the interaction of ROS, TNF-α, and IGF-1 signaling works.

The activation of Akt is known to be sensitive to the binding of the insulin and IGF-1 receptors. The baseline level of Akt phosphorylation was approximately two-fold lower in the muscles of old rats [32]. This is consistent with the observed changes in IGF-1 production and cellular responsiveness. Studies further demonstrated that activation of Akt/mTOR signaling downstream of IGF-1 pathway is blunted in contraction-induced muscle growth when aged muscle compared to young adult muscle, which suggested the reduced ability of muscle hypertrophy [36,37].

The studies mentioned above strongly suggest that sarcopenia may be linked to a reduction in the activity or sensitivity of anabolic signaling proteins such as IGF-1 and Akt, but the exact mechanisms remain to be elucidated. Additionally, TNF-α may potentially influence this anabolic perturbation. Reduced muscle protein synthesis rates were related to increased circulating concentrations of several markers of immune activation [38]. In addition, plasma C-reactive protein, interleukin-6, and TNF-α receptor II concentrations were negatively related to mixed-muscle and MHC protein synthesis rates [16]. In old rats, the ability of leucine to stimulate muscle protein synthesis was significantly decreased compared to normal younger adults [39]. This defect was reversed when old rats were supplemented with antioxidants, suggesting that antioxidant supplementation could benefit muscle protein metabolism during aging [39]. Thus, it may be plausible to suggest that effective combined interventions to ameliorate the age-related loss of muscle mass should be designed to reduce the state of oxidative stress and chronic inflammation, and also, enhance the protein synthesis and reduce the protein breakdown promoted by IGF-1/Akt/mTOR signaling.

### 3.2. FOXO

Forkhead transcription factors encompass a large family of proteins characterized by a conserved DNA-binding domain termed the “forkhead box” (FOXO) [40]. Recent studies provide evidence that FOXO1 inhibits the function of anabolic pathways in skeletal muscle via increased expression and reduced phosphorylation of the translational repressor protein 4E-BP1 and impaired signaling *via* reductions in mTOR and Raptor levels [41]. These observations raise the possibility that, in mammalian skeletal muscle, FOXO1 may not only contribute to catabolic processes via activation of ubiquitin ligases [42], but may also repress anabolic pathways. FOXO1 may be an important therapeutic target for human diseases in which anabolism is impaired. In addition, Akt phosphorylates FOXO transcription factors to inhibit their translocation to the nucleus. However, when Akt inhibition in aged muscle allows FOXO to translocate, the expression of atrophy-related genes such as atrogin-1 and MuRF-1 is enhanced [43].

Aging-related transcription factors known to be redox-regulated include Forkhead transcription factors. Welle *et al*. found increased FOXO1 mRNA in aged muscle using standard microarray analysis [44]. Another recent study has shown that the nuclei of aged muscle contain more FOXO1 than those of young muscle [45], and another study demonstrated increased atrogin-1 mRNA in aged rats [46]. In addition, FOXO3A, another member of the FOXO transcription factor family, was among proteins constituting the molecular signature of sarcopenia [47]. Thus, the FOXO proteins may very well play a role in the loss of muscle mass or muscle nuclei with aging.

### 3.3. NF-κB

The nuclear factor κb (NF-κB) transcription factor is a major pleiotropic transcription factor that modulates immune, inflammation, cell survival, and proliferation responses[48]. NF-κB activity seems to directly regulate MyoD which is myogenic transcription factor, and probably other molecules such as MuRF1, during atrophy [49]. ROS and TNF-α both activate NF-κB. Using immobilization studies in rats and mice, direct muscle injections of either cytokines (TNF, INF-γ) or cancer cells, as well as denervation of the sciatic nerve to induce muscle wasting, researchers have shown that NF-κB levels are strongly upregulated upon muscle atrophy [50–52]. Indeed, attempts to inhibit the NF-κB pathway in several atrophy models prevented muscle degeneration and myofiber death [50,51].

Sarcopenia is a normal consequence of aging that leads to the gradual inability to maintain skeletal muscle function and mass. In one study, NF-κB protein concentrations were found to be four-fold higher in elderly human muscles compared to those of young people; this increased concentration is accompanied by anabolic signaling deficits oberserved in wasting, aging muscle [53]. Aging also affected TNF-α signaling to NF-κB. Intermediary proteins (IKKγ, IκBα, and p65), which are responsible for the transmission of the TNF-α activation of NF-κB, increased with age in the soleus muscle. Moreover, TNF-α stimulation of both inflammatory and apoptotic pathways was attenuated when CR (caloric restriction) was applied [54]. However, to date there are no documented studies that have investigated the exact mechanism by which NF-κB acts in aging muscle.

### 3.4. MAPK

Li and collegues found that the mitogen-activated protein kinases (MAPKs): p38, ERK1/2 (extracellular signal-regulated kinase 1/2), and JNK (c-Jun NH(2)-terminal kinase) were all activated in myotubes exposed to either TNF-α or H2O2 [55]. The ERK1/2 pathway can activate several substrates, such as p90RSK (p90 ribsosomal S6 kinase), leading to the activation of transcription factors and the ribosomal subunit S6. ERK1/2 can also activate kinases associated with protein translation such as Mnk 1 (MAPK-interacting kinase 1) and its downstream substrate, eukaryotic initiation factor 4E (eIF4E) [56]. One study recently found that the higher baseline levels of ERK1/2, p90RSK and Mnk 1 in aged muscle compared to young muscle, possibly a compansory mechanism by the skeletal muscle with increasing age, trying to increasing protein synthesis [57]. In addition, this study found that aged muscle had a decrease in ERK1/2, p90RSK, Mnk 1, p38MAPK and JNK/SAPK phosphorylation after a bout of exercise. This study was the first to provide evidence that MAPK proteins are differentially activated at rest and in response to a bout of resistance exercise in the skeletal muscle of young and old men [58]. More recently, it has been found that unresponsive or decreased ERK 1/2 after resistance exercise and supplementation of essential amino acids in aged muscle may be play a role in the delayed activation of muscle protein synthesis [59].

Moreover, p38MAPK signaling has been shown to promote the expression of atrogin-1 in myotubes [60]. Preliminary evidence implicating JNK as a mediator of ROS-induced apoptosis suggests a link between ROS, JNK, and apoptosis [61], but how JNK mediates ROS-induced apoptosis needs to be determined. Recent data indicate that caspase 2 and JNK-mediated intrinsic pathway signaling constitute one of the mechanisms involved in the age-related increase in muscle cell apoptosis [62].

### 3.5. MuRF1 and Atrogin-1

The muscle ubiquitin–proteasome system has been shown to mediate a large part of the degradation of short-lived proteins or long-lived myofibrillar proteins in skeletal muscle [63]. Major advances have been made recently in the elucidation of signaling pathways that regulate the muscle ubiquitin–proteasome system. The addition of ubiquitin to a protein substrate is believed to be an exquisitely regulated process. This process requires three distinct components: an E1 ubiquitin-activating enzyme, an E2 ubiquitin-conjugating enzyme, and an E3 ubiquitin-ligating enzyme. The E3s play an important role in determining which proteins are targeted for degradation by the proteasome. Two muscle-specific E3s called atrogin-1/MAFbx and MuRF1 that are overexpressed in numerous catabolic states have been identified [64]. Mice in which either enzyme is knocked out were partially resistant to muscle atrophy [65]. The transcription of atrogin-1/MAFbx is under the control of FOXO [66], whereas MuRF-1 transcription is driven by the activation of NF-κB [67]. Conversely, constitutive activation of Akt by genetic manipulation was shown to be sufficient to block the atrophy-associated increases in MAFbx and MuRF1 transcription that are associated with the inhibition of FOXO transcription factors [66].

However, in aging human and rodent muscle there are data showing increased [68], decreased [69] and unchanged gene levels [70] of these E3 ligases, indicating the complexity of the regulation of protein breakdown in aging muscle. These findings suggest that older sarcopenic individuals do not have as robust of a proteolytic program as has been reported in induced atrophy models. Perhaps the less robust proteolytic program is related to the rate of muscle loss because humans gradually lose muscle mass over a period of decades compared to days or weeks in the atrophy models of rodents [70]. The age difference between the studies may have impacted the findings because individuals >80 years old have a greater prevalence of sarcopenia, and more severe muscle atrophy compared to individuals only a decade younger [71]. Collectively, these research studies point to differences in basal proteolytic gene induction that may be related to the degree of muscle mass loss.

### 3.6. PGC-1α

The transcriptional coactivator peroxisome proliferator-activated receptor γ coactivator-1α (PGC-1α) may mediate the important effects of exercise in human health to prevent muscle catabolism and muscle wasting by several mechanisms, including regulation of mitochondrial content and oxidative metabolism and suppression of chronic inflammation and muscle catabolism [72]. PGC-1α plays a leading role in regulating several properties that are responsible for the protection and maintenance of mitochondrial function in healthy muscle, including antioxidant protection, mitochondrial biosynthesis, and type I fiber determination [73]. The anti-muscle wasting effect of PGC-1α may be due to the reduction of atrophy-specific gene transcription by inhibition of FOXO3 activity [74], increases in the gene program for protein synthesis, and stabilization of the postsynaptic side of the neuromuscular junction (NMJ) [75]. It has previously been shown that PGC-1α has a powerful suppressive effect on ROS production that is in parallel with its effects in elevating mitochondrial respiration. This effect is due to the PGC-1α-mediated expression of genes involved in ROS detoxification, as well as the expression of uncoupling proteins that can attenuate ROS production [76].

The signaling-transcription network that is responsible for exercise training-induced PGC-1α gene transcription and enhanced mitochondrial biogenesis remain to be identified. It is likely associated with endurance exercise-induced changes in calcium signaling and the AMP/ATP ratio in skeletal muscle, which activate several important transcription factors [77], including the cAMP responsive element binding protein (CREB), the myocyte enhancer factors 2 (MEF2C and MEF2D), the nuclear factor of activated T cells (NFAT), and AMP-activated protein kinase (AMPK) [78].

Downregulation of the gene transcription of the components of the electron transport chain across a range of tissues underlies the reduced mitochondrial biosynthesis seen during aging, the key to which is PGC-1α. The impaired ability of the aged cells to induce coactivators of mitochondrial gene transcription, such as PGC-1α [79], may be the key to the downregulation of biosynthesis and the increased dysfunction of mitochondria with age. However, the mechanisms for the reduced expression of PGC-1α remain to be elucidated.

In contrast, PGC-1α is found to be elevated in chronically exercised skeletal muscle, even between individual bouts of exercise, when compared to untrained muscle, which may be the adaptation of skeletal muscle to endurance exercise [73]. It is, therefore, plausible to suggest that the increased density and function of mitochondria and the suppression of ROS generation and chronic inflammation in muscle via exercise-mediated induction of PGC-1α gene expression should lower the frequency and/or severity of age-related muscle mass loss [80].

## 4. Apoptosis Signaling

Only recently has apoptosis been addressed as a possible mechanism contributing to the aging process and the development of age-related muscle loss [81]. Apoptosis is a highly regulated form of cell death that is characterized by specific morphological, biochemical, and molecular events. Several apoptotic stimuli exist, including calcium [82], oxidative stress [83], and TNF-α [54], which can initiate apoptotic signaling in aged skeletal muscle. It has recently been reported that the number of TUNEL (terminal deoxynucleotidyl transferase dUTP nick end labeling) positive cells increases significantly in older adults with reduced muscle strength, indicating a preferential role for apoptosis in the reduction of muscle function with age [70].

Recent data suggest that age-related sarcopenia and muscle fatigability are associated with enhanced ROS production, increased mitochondrial apoptotic susceptibility, and reduced transcriptional drive for mitochondrial biogenesis (e.g., lower protein levels of PGC-1α) [79]. Mitochondrial dysfunction may trigger the initial events of mitochondrial mediated apoptosis via the release of proapoptotic proteins into the cytosol [79]. Moreover, in very old age the mitochondrial caspase-independent apoptotic pathway (apoptosis inducing factor, AIF; endonuclease G, Endo G) may play a more prominent role in skeletal muscle loss than caspase-mediated apoptosis (cytochrome c, Bax/Bcl2) [21].

In addition, there is evidence that cytosolic Ca^2+^ levels increase with age [82], providing a favorable environment for the activation of the endoplasmic reticulum-mediated apoptotic pathway. However, there is little evidence for the activation of this pathway. Nevertheless, aging is associated with increased DNA fragmentation, cleaved caspase-3 in rat skeletal muscle [81]. Additionally, recent results indicate that caspase-2 and JNK-mediated intrinsic pathway signaling activated by calcium and oxidative stress are involved in the age-related increase in muscle cell apoptosis [62].

Finally, increased levels and production of TNF-α by aged skeletal muscle [54] may act as a signal to activate death receptors on the cell surface membrane. Recent data [54] demonstrated that life-long caloric restriction reduced markers of apoptosis induced by TNF-α in aging rat skeletal muscle. Moreover, treadmill exercise training [84] and resistance training [85] can attenuate both fiber atrophy and pro-apoptotic signaling in aging skeletal muscle.

## 5. Considerations for Combined Interventions

### 5.1. Exercise

Endurance exercise enhances muscle metabolism protein synthesis and mitochondrial biogenesis [86]. Endurance exercise may mediate its anti-inflammatory and anti-atrophy effects by many routes, including the upregulation of PGC-1α in muscle [87], downregulation of Toll-like receptors [88], and enhanced release of IL-6 resulting in inhibition of TNF production [89]. Treadmill exercise training attenuates fiber atrophy and pro-apoptotic signaling in aging skeletal muscle [84].

It is resistance exercise that promotes muscle hypertrophy in young and middle-aged individuals [90]. It has been recently demonstrated that resistance training three days/wk led to more robust hypertrophy in young *vs.* old participants, particularly among men [91]. Moreover, animal models using both genetic manipulation and exercise training show that IGF-1/Akt/mTOR signaling is a key factor in mediating the adaptive responses of skeletal muscle to resistance exercise [28]. In fact, resistance training can increase the activity of mitochondrial enzymes [92], and decreases skeletal muscle TNF-α in frail elderly humans [85].

It should be taken into consideration that there are hundreds of muscles of different types in human and rodent body, each of which displays different degrees of atrophy during the aging process. The different types of muscles may require combined resistance and endurance training to activate or inhibit some major signaling mechanisms to combat age-related loss of muscle mass. More interestingly, combined resistance and endurance training is of greater value than either type alone in optimizing body composition and/or improving physical fitness in older men, although the mechanisms are unknown [93]. However, it is worth noting that simultaneous training for both strength and endurance results in a compromised adaptation compared to training with either exercise mode alone. This effect has been variously described as the concurrent training effect or the interference effect [94]. It now appears that the genetic and molecular mechanisms of adaptation induced by resistance- and endurance-based training are distinct, with each mode of exercise activating and/or repressing specific subsets of genes and cellular signaling pathways [95]. We therefore need to know more about the compatibility or incompatibility of the two pathways involving PGC-1α and Akt in muscle adaptation to various types of exercise training. This knowledge would allow the design of optimal exercise interventions targeting sarcopenia.

### 5.2. Caloric Restriction (CR)

The second consideration is whether exercise needs to be combined with antioxidant and anti-inflammation interventions, given that the growth and anabolic ability of aging muscle is reduced in response to exercise-induced stimuli, due to the state of oxidative stress and chronic low-grade inflammation. In fact, the effectiveness of caloric restriction in ameliorating the aging process in skeletal muscle has been extensively demonstrated [6]. Long-term caloric restriction attenuates the age-induced elevation in the production of ROS by mitochondria and oxidative damage to mitochondrial DNA (mtDNA) [96]. McKiernan *et al*. found that lifelong 40% caloric restriction results in a significant decrease in the rate of muscle mass loss and attenuates age-induced fiber loss [97].

Recent research first demonstrated that combined wheel running and mild caloric restriction significantly preserved a higher muscle mass/body mass ratio and fiber cross-sectional area, which was related to attenuation of oxidative stress and depressed IGF-1 levels [98]. However, CR alone didnot produce this effect [98]. These striking and novel findings strongly suggest that long-term primary prevention strategies in adults to address age-induced sarcopenia should include lifelong mild caloric restriction and daily, continuous voluntary exercise.

### 5.3. Combined Interventions

Malnutrution and alterations in the muscle anabolic response to nutritional stimuli have been identified as potentially preventable factors that may significantly contribute to sarcopenia [53]. Thus, nutritional interventions may be useful for the prevention and treatment of sarcopenia [99]. Recent data have demonstrated that anabolic nutrients (a leucine-enriched essential amino acid–carbohydrate mixture) increase the phosphorylation status of mTOR-associated signaling proteins in human muscle, in association with an increase in protein synthesis not only via enhanced translation initiation but also through signalling promoting translation elongation [100]. One study has also shown that branched-chain amino acids increase p70S6k phosphorylation in human skeletal muscle after resistance exercise [101]. In fact, the combination of resistance exercise and EAA (essential amino acid) ingestion has been demonstrated as a useful strategy to combat sarcopenia [59].

Other nutrition factor should also be considered, including the dietary antioxidant such as carotenoids, which contribute to reduce the state of oxidative stress and chronic inflammation [10]. Antioxidant supplementation could benefit muscle protein metabolism during aging [39], but further studies are needed to determine the mechanism involved and to establish if it could be a useful nutritional tool to slow down sarcopenia with longer supplementation.

Moreover, the synergistic effects of CR with maintained protein intake may help to limit the progression of sarcopenia by optimizing the turnover rates and functions of major proteins in skeletal muscle, to improve the synthesis rate of myosin and actin and grip force, to decrease mitochondrial protein oxidative damage, and to enhance mitochondrial biogenesis [102].

**Figure 2.**
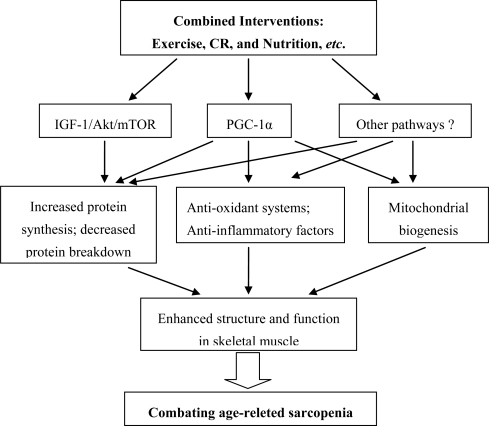
Hypothetical scheme for how combined interventions can affect sarcopenia.

Finally, given the complexity of age-related loss of muscle mass and function, it is perhaps the combination of three or more factors that will become the focus of future studies designed to maintain skeletal muscle mass and function. An interesting combination that should be considered is combined exercise, CR, and nutrition, which would likely produce more additive or interactive effects that may improve the structure and function of muscle, as seen in Figure 2. This strategy remains to be tested.

An interesting combination that should be considered is combined exercise, CR, and nutrition, which would likely improve the structure and function of muscle through multipli mechanisms induced by activation of IGF-1/Akt/mTOR, PGC-1α, and/or other pathways unidentified for combating age-related sarcopenia, including increased protein metabolism, redox balance, mitochondrial biogenesis, and anti-inflammatory ability. This strategy remains to be tested.
